# Portable and Affordable Light Source-Based Photoacoustic Tomography

**DOI:** 10.3390/s20216173

**Published:** 2020-10-29

**Authors:** Mithun Kuniyil Ajith Singh, Wenfeng Xia

**Affiliations:** 1 Research and Business Development Division, CYBERDYNE INC., Stationsplein 45, A4.004, 3013 AK Rotterdam, The Netherlands; mithun_ajith@cyberdyne.jp; 2 School of Biomedical Engineering& Imaging Sciences, King’s College London, King’s Health Partners, St Thomas’ Hospital, London SE1 7EH, UK

**Keywords:** photoacoustic imaging, photoacoustic computed tomography, light-emitting diodes, laser diodes

## Abstract

Photoacoustic imaging is a hybrid imaging modality that offers the advantages of optical (spectroscopic contrast) and ultrasound imaging (scalable spatial resolution and imaging depth). This promising modality has shown excellent potential in a wide range of preclinical and clinical imaging and sensing applications. Even though photoacoustic imaging technology has matured in research settings, its clinical translation is not happening at the expected pace. One of the main reasons for this is the requirement of bulky and expensive pulsed lasers for excitation. To accelerate the clinical translation of photoacoustic imaging and explore its potential in resource-limited settings, it is of paramount importance to develop portable and affordable light sources that can be used as the excitation light source. In this review, we focus on the following aspects: (1) the basic theory of photoacoustic imaging; (2) inexpensive light sources and different implementations; and (3) important preclinical and clinical applications, demonstrated using affordable light source-based photoacoustics. The main focus will be on laser diodes and light-emitting diodes as they have demonstrated promise in photoacoustic tomography—the key technological developments in these areas will be thoroughly reviewed. We believe that this review will be a useful opus for both the beginners and experts in the field of biomedical photoacoustic imaging.

## 1. Introduction

Photoacoustic imaging (PAI) is an emerging biomedical imaging modality that is based on the detection of ultrasound (US) waves generated from tissue in response to the absorption of temporally varying optical energy [1,2,3,4,5]. Typically, in PAI, short-pulsed or temporally modulated light is delivered to a tissue surface, and the light then propagates diffusively through the tissue during which a portion of the optical energy is selectively absorbed by various light absorbing structures, such as endogenous tissue chromophores and exogenous contrast agents [2]. These endogenous tissue chromophores include DNA/RNA, oxy- and deoxy-hemoglobin, lipid, and melanin; exogenous contrast agents include small molecular dyes, organic nanostructures, metal and carbon nanoparticles, and genetically encoded chromophores [3]. The absorption of the time-varying optical energies leads to rapid and subtle local temperature rises, and subsequently, the generation of broadband US waves in the MHz frequency range. These initial pressure waves propagate outwards and can be received by US detectors at the tissue surface to form images of the absorbing structures. Since optical absorption spectra of common tissue chromophores (hemoglobin, melanin, lipid, etc.) are well known, it is possible to tune the light excitation wavelength and functionally characterize the tissue using multispectral PAI [1]. For example, one can quantitatively detect oxygen saturation inside a blood vessel with high spatial and temporal resolution at depths up to a few centimeters, which is unachievable using any other imaging modalities. PAI involves US detection and it is straight forward to implement this technique in conventional US imaging equipment, offering structural, functional, and molecular imaging details in a single image acquisition [6]. PAI is undoubtedly one of the fastest growing research-based medical imaging modalities in recent times. However, one of the key factors hindering the clinical translation of this promising technique is the requirement of bulky and expensive solid-state lasers for tissue illumination [7]. In this review, after covering the principles of PAI, we will focus on affordable light sources (light-emitting diodes and laser diodes) that are being explored as alternative excitation sources for photoacoustic (PA) tomography, demonstrate its applications, and also discuss the advantages and disadvantages of different illumination sources in PAI. To focus more on PA tomography (PAT) using light-emitting diodes (LEDs) and laser diodes (LDs), we exclude developments and applications demonstrating PA microscopy and endoscopy.

## 2. Principles of Photoacoustic Imaging

**Figure 1:**
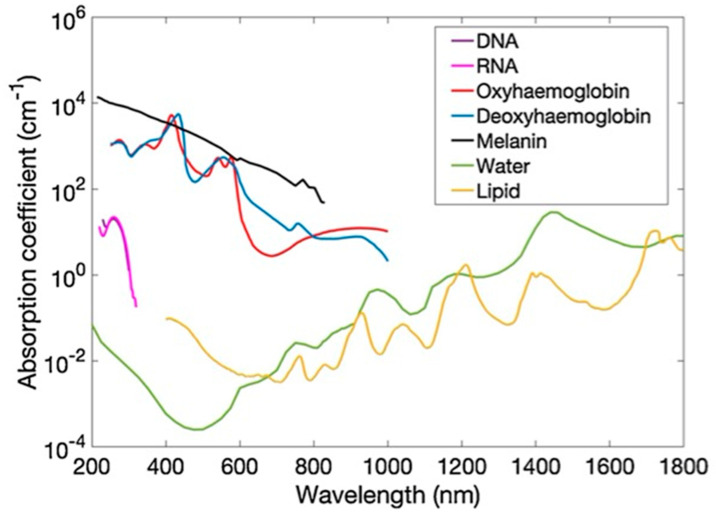
Absorption coefficient spectra (µa) of endogenous tissue chromophores, including DNA, RNA, oxyhemoglobin, deoxyhemoglobin (150 g L^−1^), melanin, water, and lipid. Adapted with permission from T. Zhao, A. E. Desjardins, S. Ourselin, T. Vercauteren, W. Xia, Photoacoustics, Vol.16, Article ID100146, 2019; licensed under a Creative Commons Attribution (CC BY) license.

Optical tissue imaging utilizes spectroscopic features of light–tissue interactions, such as scattering, absorption, and polarization. Since many tissue molecules possess signature optical properties (especially absorption), this interaction is quite powerful in characterizing tissue at functional and molecular levels. Figure 1 shows the optical absorption spectra of prominent tissue chromophores, such as hemoglobin, melanin, lipid, and water. The rich spectroscopic contrast of these molecules and their relations to a spectrum of diseases make optical imaging very useful [1,2,3]. However, purely light-based imaging techniques suffer from poor resolution at depths larger than a few mm, due to high scattering in the tissue [1]. While in US imaging, the tissue is insonified using sound waves and echoes are used to generate acoustic reflectivity maps [8]. This is one of the most popular medical imaging modalities with several advantages, including a high portability, affordability, accessibility, and spatial and temporal resolution. Even though US imaging offers tissue anatomical information in real time, it can provide insufficient contrast for soft tissues, and insufficient sensitivity for differentiating malignant and benign abnormalities in deep tissue [6,9].

### 2.1. Generation of Photoacoustic Signals

**Figure 2:**
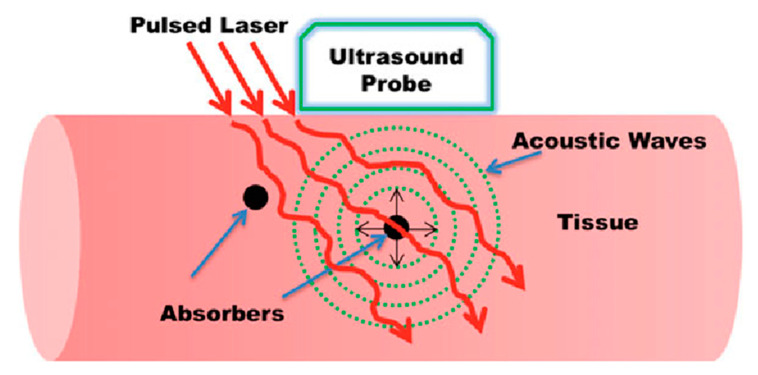
Basic principle of biomedical photoacoustic imaging. Reproduced with permission from Handheld Probe-Based Dual Mode Ultrasound/Photoacoustics for Biomedical Imaging. In: Olivo M., Dinish U. (eds) Frontiers in Biophotonics for Translational Medicine. Progress in Optical Science and Photonics, vol 3. Springer, Singapore (2016). Copyright 2016 Springer, Singapore.

The basic idea of the photoacoustic effect was described by Alexander Graham Bell long back in 1880 [10]. When pulsed light is shone on a sample that absorbs a fraction of the incident energy, the optical absorption will result in a temperature rise, leading to thermoelastic expansion of the absorbing object. This sudden pressure rise propagates as a sound wave, which then can be detected using conventional US transducers. By detecting the pressure waves, one can localize their sources (i.e., where the light was absorbed) and obtain important functional and molecular information about the studied sample [1]. The basic idea of PA imaging is schematized in Figure 2.

To generate broadband PA signals (sound waves) in the sample efficiently and obtain high-resolution images, the light pulse must be shorter than both the thermal relaxation time $\tau_{th}$ (thermal confinement) and the stress relaxation time $\tau_{s}$ (stress confinement), respectively, defined by Equations (1) and (2) [1,11].

(1) $$\tau_{th}=\frac{d_{c}^{2}}{\alpha_{th}}\;$$

where $d_{c}$ is the characteristic dimension of the structure of interest and $\alpha_{th}$ is the thermal diffusivity.

(2) $$\tau_{s}=\;\frac{d_{c}}{v_{s}}\;$$

where $v_{s}$ is the speed of sound in the tissue (~1540 m/s).

Let us consider a 15 µm optically absorbing structure inside the tissue. In this case, $\tau_{th}$ and $\tau_{s}$ will be 1.4 × 10^−3^ s and 1 × 10^−8^ s, respectively. Thus, it is clear that the pulse width of the tissue illumination source must be shorter than 10 ns to spatially resolve this 15 µm structure in a PA image. It is also important to note that the detection bandwidth of the US probe also must be high to achieve such high-resolution images, which will be discussed in greater detail later in Section 2.4. The optically induced initial pressure distribution $p_{0}$ can be estimated as

(3) $$p_{0}\left(r\right)\;=\;\Gamma \;{µ}_{a}\;\left(r\right)F\left(r\right)\;$$

where *r* refers to a spatial location within the heated volume, µ*_a_* is the optical absorption distribution of the absorbing structure, *F* is the local fluence, Γ is the Grüneisen coefficient, and *a* the dimensionless thermodynamic constant that defines the conversion efficiency of heat energy to pressure, and can be expressed as

(4) $$\Gamma =\beta v_{s}{}^{2}/C_{p\;}\;$$

where β is the isobaric volume expansion coefficient and $C_{p}$ is the specific heat at a constant pressure. It can be seen that $p_{0}$ depends on a number of parameters. Although several studies have shown that Γ can be a source of PA contrast in tissue [12], PA image contrast is generally assumed to be dominated by the local optical absorption ${µ}_{a}$ and the local fluence F, which is a product of optical absorption and scattering. Considering the strong light attenuation in the tissue, *F* decreases exponentially with tissue depth; however, optical fluence variations at a given depth in the tissue are usually much smaller compared to those of ${µ}_{a}$. As such, PA imaging is often considered as an imaging modality that is based on optical absorption of the tissue; however, it is important to note that the PA image signal is not directly proportional to ${µ}_{a}$.

### 2.2. Illumination Sources

The characteristics of the illumination sources mainly define the quality of the PA images and contribute to the high cost of the whole PAI system. PAI systems conventionally use solid-state lasers, such as Q-switched Nd: YAG lasers coupled with optical parametric oscillators (OPO). These lasers usually offer pulse widths in the range of 5–20 ns, which is very much suitable for high-resolution PAI. However, these bulky laser sources are very expensive and are not suitable for use in a clinical setting (for example in an operating room or an outpatient clinic) [13,14,15,16,17]. Recently, there has been significant research in the area of affordable light sources for PAI. Laser diodes (LDs) and light-emitting diodes (LEDs) are the most common PAI illumination sources, which offer high portability, affordability, and energy efficiency [13,14,15,16,17]. Solid-state lasers, LDs, and LEDs have completely different characteristics (pulse width, pulse repetition rate (PRR), optical output, and cost) and have their own advantages and disadvantages. Table 1 shows a comparison of the light sources used in PAI.

### 2.3. Optical Absorption

As shown in Figure 1, intrinsic optical absorbers in the tissue possess the signature absorption characteristics and the spatial distributions of these absorbers can be quantified in PAI with a high spatial resolution and large imaging depths [1]. For example, PAI offers excellent contrast for blood. Since oxyhemoglobin and deoxyhemoglobin have different optical absorption spectra, careful selection of the excitation wavelengths (even two wavelengths with good absorption difference would suffice) can help in obtaining oxygen saturation maps of the tissue with an unprecedented resolution (micro-vasculature level) [16]. Apart from intrinsic absorbers, it is feasible to image exogenous contrast agents by selecting the light wavelengths based on the absorption peak of them [17]. Considering the low absorption of water and high absorption of blood, near infrared (NIR) wavelengths are most commonly used in PAI for achieving higher imaging depths.

### 2.4. Ultrasound Propogation and Detection

The velocity of the US waves in tissue is considered constant at 1540 m/s in general. Variations are typically less than 10%, and are usually not accounted for, unless the sample is highly heterogenous. Acoustic scattering in tissue is roughly three orders of magnitude lower than optical scattering and this is the key reason for a higher imaging depth in PAI compared to other optical imaging modalities that rely on optical focusing [1,2,3,4,5]. One important factor to consider acoustically is the attenuation in the tissue, which can be modelled as below.

(5) $$\alpha =af^{b}\;\left[\mathrm{dB}/\mathrm{cm}\right]\;$$

where *a* is a tissue dependent constant, *b* is a power law factor whose value is usually ranged between 1 and 2 in the soft tissue, and *f* is the frequency expressed in MHz. In general, for soft tissue, α is considered as 1 dB cm^−1^ MHz^−1^ on average; it can reach as high as 20 dB cm^−1^ MHz^−1^ for bone (assuming *b* = 1 in both cases). As such, the characteristics of the frequency-dependent acoustic attenuation are similar to those of a low pass filter so that higher frequency signals are attenuated more in tissue. Although the PA signals can be extremely broadband with frequency contents ranging from several tens of kHz to several tens of MHz, depending on the size of the object, frequency-dependent acoustic attenuation in tissue has limited the maximum frequency content that can arrive at the detector side and thus fundamentally limits the achievable spatial resolution. The spatial resolution of a PA tomography system also depends on other factors, including the frequency bandwidth of the US detector, the active surface area of the detector element, number of detector elements, detector aperture, and image reconstruction algorithms. Moreover, as the acoustic sensitivity of an US detector decreases with the increase in frequency (which largely determines the spatial resolution), there is a trade-off between the imaging depth and spatial resolution. For example, according to a recent study by Xia et al. [18], the measured axial resolution using resolution targets immersed in water for a PAI system with a linear array US probe (central frequency: 9 MHz; −6 dB bandwidth: 77%; active surface area: 5 mm × 0.3 mm; number of element: 128; and detector aperture: 38.4 mm) was 0.22 mm and remained consistent over a depth range of 13 to 36 mm; the lateral resolution depended on the spatial location, ranging from 0.35 mm to 0.76 mm over the same depth range.

Linear array piezoelectric probes are commonly used as US detectors for PAI similar to those used for conventional US imaging. As such, real-time interleaved US and PA imaging can be performed with the same US detector and DAQ electronics [6]. US detection in PAI mode is the same as that in the US mode, except that in this case no US transmissions are performed. This dual modality approach offers many advantages involving complementary contrast and easiness in clinical translation. US imaging is a well-accepted imaging modality and it would be easier for clinicians to accept PAI as an additional technique along with conventional US imaging [7]. Typical frequencies for US imaging are in the range of 1–25 MHz. For utilizing the full benefits of PA imaging, it is important to develop new US probes with a higher bandwidth and sensitivity as the PA signals are usually broadband and their amplitudes are much lower than the pulse echo signals in US imaging. There have been significant developments in this area recently [6]. Acoustic waves can be detected using single-element transducers (and employing scanning) or using an array of elements as in conventional US probes (planar, cylindrical, or spherical) to enable 2D or 3D imaging.

### 2.5. Image Reconstruction

PAI involves the detection of acoustic signals generated by optical absorption, and image reconstruction strategies are thus stemmed from both optical and US imaging. Basic PA image reconstruction aims to retrieve the spatial distribution of the initial PA pressure or locations of the absorbed optical energy [11]. This is usually termed the acoustic inversion problem and uses various algorithms that originate from the US and sonar world. From the distribution of the initial PA pressure, it is desired to reconstruct a distribution of the optical absorption coefficient, especially when multiple wavelengths are used for PA excitation; however, this is a non-linear problem due to the wavelength-depended optical attenuation of the tissue, which is termed the optical inversion problem. Reconstruction of the optical absorption coefficient is usually termed quantitative PA imaging [11].

**Figure 3:**
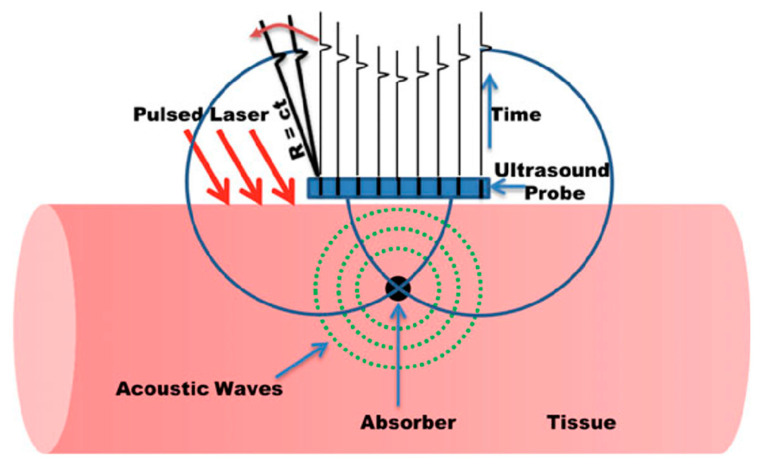
Back-projection PAI reconstruction for a planar detection geometry. Reproduced with permission from Handheld Probe-Based Dual Mode Ultrasound/Photoacoustics for Biomedical Imaging. In: Olivo M., Dinish U. (Eds) Frontiers in Biophotonics for Translational Medicine. Progress in Optical Science and Photonics, volume 3. Springer, Singapore (2016). Copyright 2016 Springer, Singapore.

There has been extensive research in the area of PA image reconstruction and different methods have been reported. Time reversal, Fourier domain analysis, analytic back-projection, radon transfer, and model-based algorithms are some of the most commonly applied methods. Kuchment et al. reported a detailed review about the different PA-based image reconstruction methods [19]. Based on the type of acoustic detection probes (linear arrays, curved arrays, etc.) and the computational capability, one can choose the right image reconstruction algorithm. Considering the ease in implementation and speed offered, back-projection is the most commonly used image reconstruction technique for processing PA data [1]. Figure 3 illustrates the concept of back-projection algorithms when the PA detection geometry is linear/planar. In this case, each detector element at a specific location records the PA data using the speed of sound (c) and time of flight (t), and then the recorded time-resolved signals are back-projected over a spherical surface of radius R = ct into the imaging volume [1].

This method is comparable to the conventional delay-and-sum beamformer, which is the most common algorithm employed in US imaging using phased arrays. It is important to note that a US-based delay-and-sum reconstruction algorithm can be used for PAT data by reducing the time-of-flight to half (PA data traverse half the time in tissue when compared to US signals). This offers the possibility to switch between these two image reconstruction modes at a high speed, which is a key requirement in handheld dual-mode PA/US imaging systems. However, the image quality offered by such an approach is not up to the mark. To solve this issue, there have been tremendous developments in the area of frequency domain algorithms, offering superior image quality with computing speeds suitable for systems suitable for real-time imaging [20,21].

## 3. LED-Based Photoacoustic Tomography

In recent years, there have been significant developments in the use of high-power LEDs in biomedical PAI, especially for superficial imaging applications [13,22]. In this section, we review the developments in this area after discussing briefly the characteristics of the LEDs and its advantages and disadvantages.

### 3.1. High-Power LEDs Suitable for Photoacoustic Tomography

An LED is a semiconductor device (P–N junction) that emits light when an electrical current passes through it. Free electrons are the majority charge carriers in N-type semiconductors. On the other hand, holes are the main charge carriers in P-type semiconductors. A P–N junction is formed when the N-type and P-type semiconductors are joined together. When a voltage is applied across the P–N junction diode (forward bias), holes (from P-substrate) and electrons (from N-substrate) move towards the junction, resulting in plenty of electrons and holes in the junction region. This fosters a strong electron–hole radiative recombination and consequent emission of photons. In case of LEDs, the radiative recombination process is dependent on spontaneous emission in which electrons in a high energy state (E_2_) move down to a lower energy state (E_1_) and combine with the available holes. The difference in energy between these two states (E_g_ = E_2_ − E_1_) results in spontaneous emission of photons in random directions [23].

### 3.2. High-Power LEDs—Technical Aspects

#### 3.2.1. Emission Wavelength

**Figure 4:**
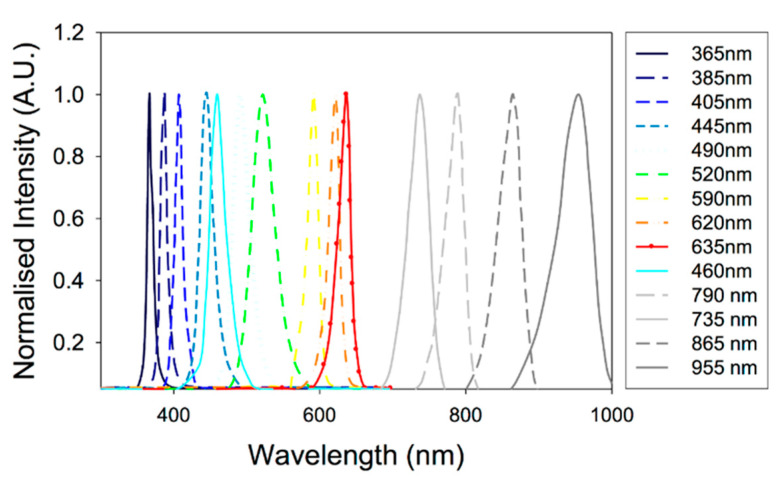
Optical spectra for a range of commercially available LEDs. Reproduced with permission from T. J. Allen and P. C. Beard, Biomedical Optics Express, Vol.7, Article ID1260, 2016; licensed under a Creative Commons Attribution (CC BY) license.

When considering the biomedical imaging applications, a key advantage of LEDs over LDs is the availability of a wide range of wavelengths. As shown in Figure 4, LEDs are available even in the visible wavelengths in which the optical absorption of hemoglobin is quite high [23].

Materials used for designing the semiconductor (P–N junction) and its energy bandgap (E_g_) are the factors that define the emission wavelength of the LEDs. Thus, one can tune the emission wavelength (visible to NIR range suitable for PAI) of LEDs by carefully selecting the semiconductor material [23]. For example, NIR wavelengths, the most suitable wavelengths for deep-tissue PAI can be developed by using aluminum gallium arsenide (AlxGa1-xAs). By changing the mole fraction (x) of the aluminum (Al) in AlxGa1-xAs, it is straightforward to tune the wavelength range from 624 nm to 920 nm. For obtaining wavelengths in the visible range (570–650 nm), aluminum gallium indium phosphide ((AlxGa1-x)0.5In0.5P) is the right candidate for the semiconductor material. In this case, by increasing the mole fraction (x) of the aluminum (Al), one can reduce the emission wavelength. For even shorter wavelengths (440–550 nm), it is recommended to use indium gallium nitride (InGaN) as the semiconductor material. An increase in indium (In) will result in shifts of emission from shorter to longer wavelengths. The materials used to design LEDs with different wavelengths are summarized in Table 2. With the availability in a wide range of wavelengths, LED illumination is very well suited for multispectral PAI [22].

#### 3.2.2. Overdriving LEDs

LEDs are designed to be used for continuous wave (CW) operation and its rated current is valid in this mode. However, it is possible to operate LEDs in the pulsed mode by driving it with higher current pulses of lower duty cycles (less than <0.1%). This way, one can safely increase the optical output and use these affordable devices for PAI [22,24]. Even though LED manufacturers do not provide specifications for pulsed operation, recent studies have demonstrated that it is safe to drive LEDs using a higher current (ten times their rated current) at a duty cycle lower than 0.1% without any noticeable damage [24].

**Figure 5:**
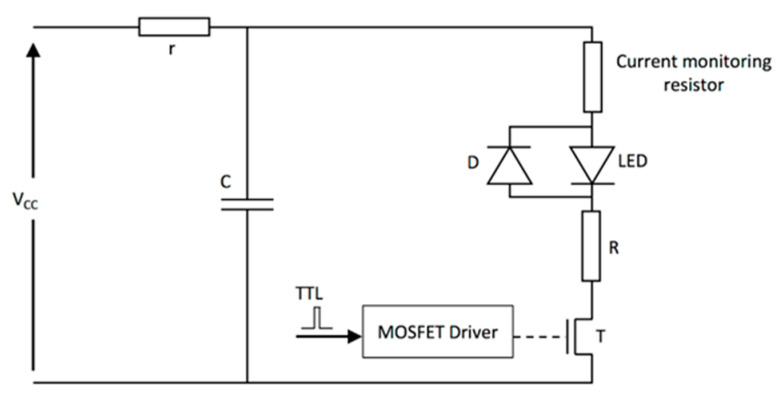
Schematic of a typical LED driver for the pulse operation mode. Vcc is the voltage provided by the power supply, T is the transistor used to switch the LED on and off, C is the storage capacitor, R is the limiting resistor, r is the charging resistor, D is a diode, and MOSFET is a metal oxide semiconductor field-effect transistor. Reprinted with permission from High-Power Light Emitting Diodes; An Alternative Excitation Source for Photoacoustic Tomography. In: Kuniyil Ajith Singh M. (eds) LED-Based Photoacoustic Imaging. Progress in Optical Science and Photonics, vol 7. Springer, Singapore. Copyright 2020 Springer, Singapore.

To operate LEDs in the pulsed mode, special electronic drivers are required. Pulsed LED drivers are most commonly composed of a capacitor, which is used as a storage element that discharges through the LED when a fast-switching device (metal oxide semiconductor field-effect transistors (MOSFETs) are used commonly) is activated. Figure 5 shows a schematic of a typical LED driver.

#### 3.2.3. Optical Output Power

**Figure 6:**
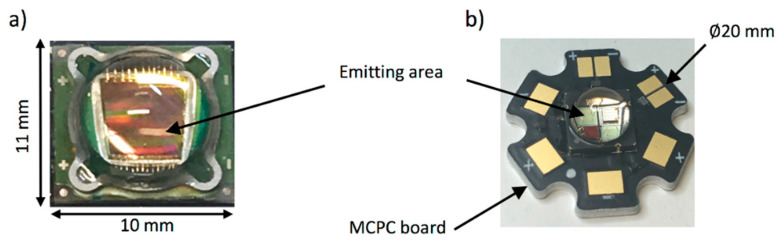
Photographs of high-power LEDs: (**a**) high-power LED (SST-90) with an emitting area of 9 mm^2^; (**b**) high-power multi-wavelength LED (LZ4-00MC00, LedEngin, Inc., California, USA), composed of 4 LEDs emitting at 452, 520, 520, and 618 nm, each with a 1 mm^2^ emitting area and mounted on a metal-core printed circuit (MCPC) board. These devices are encapsulated in spherical glass lenses. Reprinted with permission from High-Power Light Emitting Diodes; An Alternative Excitation Source for Photoacoustic Tomography. In: Kuniyil Ajith Singh M. (Eds) LED-Based Photoacoustic Imaging. Progress in Optical Science and Photonics, vol 7. Springer, Singapore. Copyright 2020 Springer, Singapore.

In its default continuous wave mode, typical high-power LEDs can provide optical output up to a few watts, which is dependent on the wavelength and also the size of the elements [24]. It is essential to use the LED elements within its allowed current rating (usually around 1 A). For avoiding heating issues, high power LED elements are usually mounted on heatsinks. However, it is critical to consider the amount of heat generated, especially when the goal is to integrate a light source and US probe in a single housing for handheld PA and US imaging. Photographs of two representative high-power LEDs are shown in Figure 6, where Figure 6a shows a device with a large emitting area (9 mm^2^) and Figure 6b shows a multi-wavelength device composed of 4 LED elements, each with a smaller emitting area (1 mm^2^) and at a different wavelength and mounted on a metal-core printed circuit (MCPC) board for efficient heat removal.

**Figure 7:**
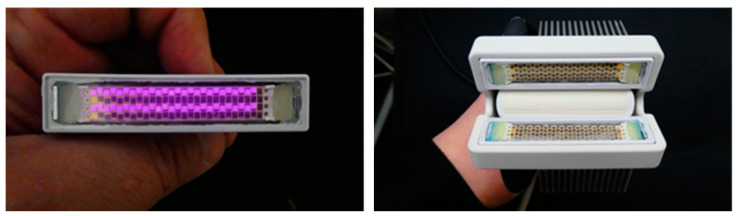
Photograph of an LED array developed by CYBERDYNE INC with four rows of LED elements, in which Rows 1 and 3 are 850 nm elements, and Rows 2 and 4 are 750 nm elements (**left**). In this picture, the 850 nm elements are activated and captured using an IR camera. Photograph of a LED-based PA/US probe developed by CYBERDYNE INC, in which two LED arrays (750/850 nm) are placed on both sides of a linear array US probe (7 MHz) (**right**).

In recent years, the possibility of using multiple LED elements as an array have also been explored to generate a higher optical output required for PAT. Figure 7 shows a photograph of an LED array with 750 nm and 850 nm LED elements arranged in an interleaved manner (left panel, 36 elements per row, 4 rows in total) and also the integrated PA and US probe in which the LED arrays are fixed on a conventional linear array US probe (right panel). These high-power LED arrays are commercialized by CYBERDYNE INC (Tsukuba, Japan) for research use [25].

#### 3.2.4. Pulse Repetition Rate (PRR)

The typical PRR of conventional solid-state lasers are in the range of 10–200 Hz. For obtaining good signal-to-noise ratios (SNRs) in deep tissue PAI, it is usually required to perform signal averaging over multiple imaging frames, leading to low frame rates in a solid-state laser-based PAI system. On the other hand, PRRs of LEDs are in the KHz range and this will help to average more frames to improve SNR, without compromising the temporal resolution. Moreover, the large PRR makes high-speed PAT a possibility. LED arrays at 850 nm with a PRR of 16 KHz have been reported by Zhu et al. recently and they demonstrated dynamic PAI using commercially available LED arrays and conventional linear array US probes [26].

#### 3.2.5. Pulse Width

The pulse width of LEDs are tens of nanoseconds (30–100 ns), whereas that of the solid-state lasers is usually less than ten nanoseconds. The temporal pulse width imposes a limit on the spatial resolution of the PAI systems [1]. For example, if an LED element with a 70 ns pulse width is used as the excitation source, the finest spatial resolution that is potentially achievable can be roughly estimated as 105 µm (= 70 ns × 1500 µm/µs). However, conventional US probes (5–7 MHz probes) have reception bandwidth limits and thus further limits its axial resolution to approximately 200–300 µm. One may generate broad bandwidth PA signals when the excitation pulse width is low (for example, 5 ns as in a solid-state laser), but detection sensitivity beyond the US bandwidth is usually very low and the spatial resolution is thus limited by the bandwidth of the US probe. It has been demonstrated that the pulse widths of LEDs are suitable for deep tissue imaging using conventional US probes [13]. A recent study also has shown that tuning the pulse width of the LEDs is feasible and this would be helpful if transducers with different center frequencies and bandwidths are used for the detection [27].

#### 3.2.6. Spatial Divergence of LEDs

Conventional high-power LEDs have larger emission angles when compared to other sources. The spatial divergence of commercially available high-power LED arrays (CYBERDYNE INC, Tsukuba, Japan) is approximately +/−60° [28,29], which is acceptable in PAT where a relatively large illumination area is usually required. However, because of the large divergence and larger emission area (~1 mm^2^), it is difficult to collimate the light and couple it to optical fibers for applications like minimally invasive PA imaging [9,30,31,32]. In terms of eye/skin safety, high spatial divergence in combination with a low optical output makes LEDs a safer alternative to solid-state lasers for non-invasive PAI [20].

### 3.3. Technical Developments in LED-Based Photoacoustic Tomography

The first report on the use of LEDs as an illumination source in PAI was from Jansen in 2011 [33]. In this work, a 627 nm LED element was used (Luxeon LXHL_PD09), which has been measured to yield approximately 250 mW of light output when supplied with 1 A DC current. Using a special electronic driver, the LED was supplied with 60-ns current pulses with a peak value of 40 A, resulting in a pulse energy of 400 nJ per pulse with a pulse width of 60 ns and a PRR of 200 Hz. Light focusing was performed to generate the fluence required for generating a PA response. To obtain PA signals from a non-realistic gelatin-based phantom, 50,000 A-lines were averaged. Owing to significant developments in the field of solid-state technology, there have been significant developments in LED technology (optical output, PRR, and pulse width), which resulted in the step-by-step development of LED-based PAI technology after 2011.

**Figure 8:**
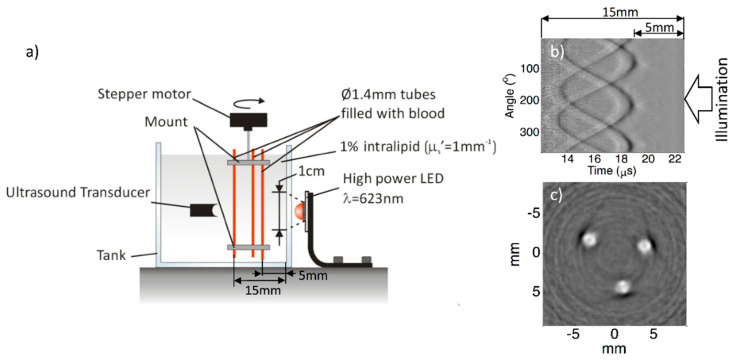
(**a**) Experimental setup; (**b**) RF PA signals of three 1.4 mm tubes filled with human blood immersed in water mixed with intralipid; (**c**) final reconstructed PA image. Optical output energy attained = 9 μJ. Number of frames averaged: 5000. Reproduced with permission from T. J. Allen and P. C. Beard, Biomedical Optics Express, Vol.7, Article ID1260, 2016; licensed under a Creative Commons Attribution (CC BY) license.

In 2013, Allen and Beard worked on this further and demonstrated that LEDs can be used as illumination sources in biomedical PA imaging [34]. In this work, they demonstrated the potential of using LEDs as an alternative excitation source in multispectral PA imaging. Recently, the same group achieved an imaging depth of 1.5 cm in tissue mimicking phantoms when using LEDs as an illumination source in PA imaging [24]. In this work, they obtained an LED output power of 10 μJ/pulse by overdriving 620 nm elements and averaging thousands of frames to improve the SNR. Figure 8a shows a schematic of the experimental setup used by them. Figure 8b shows a raw RF signal obtained and 8c shows a reconstructed image. With the same PRR of 200 Hz, as in the previous study, they achieved a frame rate that is 1000 times better, thanks to the higher optical output achieved using a MOSFET-based electronic driver.

Although these pioneering works demonstrated the feasibility of using LEDs as illumination sources in PAI, no in vivo results have been reported. The main reason behind this is the optical energy of the LEDs, which is magnitudes lower than that of a solid-state laser or even some LDs. In 2015, Agano et al. demonstrated that it was feasible to combine hundreds of high-power LED elements in a rectangular package and could be pulsed simultaneously to perform biomedical PAI [35,36,37]. Using this novel technology, a high-power LED-array-based PA imaging system (AcousticX) was commercialized by a Japanese company (PreXion Corporation, later the technology was acquired by CYBERDYNE, INC, Japan).

With AcousticX, a single LED element provided an output energy of 0.024 µJ per pulse, with a pulse duration of 70 ns and 1 A DC current. By developing LED elements with a double stack structure, arranging them in an array, and applying 20 times the rated current, a light output of 200 µJ per pulse at a wavelength of 850 nm was achieved. Two of these arrays were kept on both sides of a linear array US probe for performing real-time PA and US imaging. The repetition rate of this first commercial LED-based PAI system was 4 KHz.

**Figure 9:**
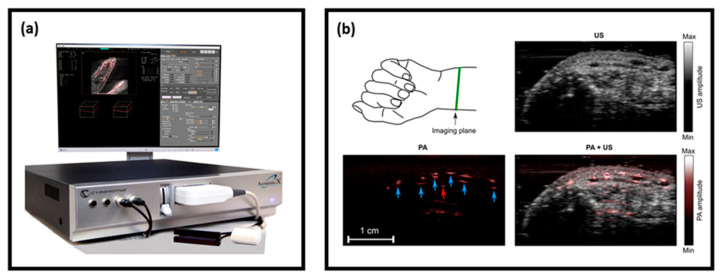
(**a**) Photograph of the LED-based PA and US imaging system—AcousticX; (**b**) US (gray colormap), PA (hot colormap), and US/PA overlay cross-section images of a human volunteer’s wrist acquired at a frame rate of 10 Hz. Blood vessels are marked using blue arrows in the PA image. Adapted with permission from W. Xia, M. Kuniyil Ajith Singh, E. Maneas, N. Sato, and A. E. Desjardins, Sensors, Vol.18, Article ID1394, licensed under a Creative Commons Attribution (CC BY) license.

The system was thoroughly characterized by several research groups for its spatial resolution and imaging depth, and subsequently its capability for functional, structural, and molecular imaging was demonstrated [26,38,39,40,41,42]. Figure 9 shows a photograph of the system and the PA and US images of a human volunteer’s wrist, which was acquired in a real-time handheld operation, demonstrating the capability of obtaining a complementary contrast (structural details from the US image and vasculature details from PA image) in a single measurement [18].

**Figure 10:**
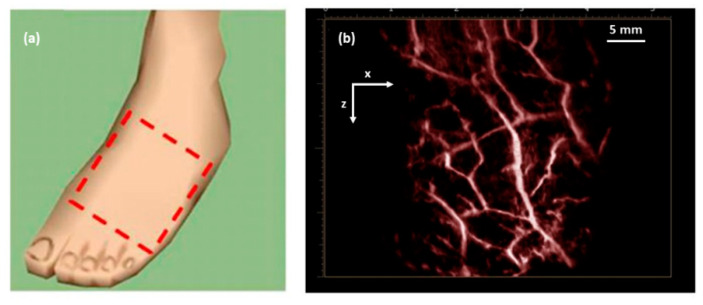
(**a**) Dashed box showing the imaging area on a human volunteer’s foot dorsum; (**b**) 3D maximum intensity projection PA image of the area marked in (**a**), clearly visualizing the vasculature network. Adapted with permission from. Reprinted with permission from Clinical Translation of Photoacoustic Imaging—Opportunities and Challenges from an Industry Perspective. In: Kuniyil Ajith Singh M. (Eds) LED-Based Photoacoustic Imaging. Progress in Optical Science and Photonics, vol 7. Springer, Singapore. Copyright 2020 Springer, Singapore.

In another study [7], utilizing the high temporal resolution of LED-based PAI, it was also possible to scan through an area of interest to generate 3D maps of the vasculature on a human volunteer’s foot dorsum, as shown in Figure 10. The total time required to generate such a high-resolution 3D PA image with a large field-of-view was less than 15 s, including scanning and image reconstruction and rendering [7].

The system has been recently used extensively by several research groups in a number of studies to explore its potential preclinical and clinical applications [43,44,45,46,47,48,49,50,51], which are summarized in Table 3. All these reports clearly demonstrated the potential of LED-based PAI in structural, functional, and molecular imaging applications in a preclinical and a clinical setting. Even though the optical output power of the LEDs or LED arrays are not comparable to a solid-state laser, it is encouraging that LED-based PAI is able to consistently achieve an imaging depth of around 5–10 mm. The main reason for this is the high PRR (~16 KHz), which allows averaging over a large number of image frames while maintaining a good temporal resolution; one can easily achieve video rates even after averaging hundreds of frames. Considering the portability, affordability, safety, and ease-of-use, LED-based PAI holds strong potential in clinical translation of PA imaging as an additional modality along with conventional pulse-echo US imaging.

## 4. Laser Diode-Based Photoacoustic Tomography

Just like LEDs, LDs are also used as an excitation source in PAT, as an alternative to bulky and expensive lasers [52]. With high optical energy (when compared to LEDs) and compactness, pulsed LDs are suitable for performing deep-tissue PAT. In this section, after discussing some of the technical details, specifications, and development of LD-based PAT, we shortly introduce the applications that demonstrate the use of this technique. For the technical aspects, we will focus on the key differences when compared to LEDs.

### 4.1. High-Power Pulsed Laser Diodes

LD is a semiconductor laser device in which the laser beam is produced at the interface region in a P–I–N (positive–interface–negative) diode region. They are electrically pumped semiconductor laser sources. They convert the input electric energy into light energy, in comparison with conventional lasers in which the input light energy from a flash lamp is converted into laser output [14]. The energy efficiency of these bulky lasers is not high, and this results in an enormous amount of heat generation (water cooling, etc., is thus required, which increases the footprint of the device). On the other hand, the energy efficiency of an LD is very high and thus a minimal amount of heat will be generated. In recent years, there have been multiple studies about the development and use of pulsed LDs in PAT [14,15,53].

### 4.2. Pulsed Laser Diodes—Technical Aspects

#### 4.2.1. Emission Wavelengths

Conventionally, continuous wave laser diodes are available in a wide range of wavelengths (visible to NIR range). However, pulsed LDs are available only in the NIR range, most probably because of the lower optical energy generated when overdriven with the short pulses required in PAI. In the NIR range, pulsed LDs offer a far higher optical energy when compared to LEDs and thus are ideal sources of illumination in multispectral PAI of deep tissue.

Even though the fundamental process of light generation of LEDs and LDs are the same, LDs generate stimulated emissions and are considered as laser sources with a high spatial coherence. Just as in LEDs, semiconductor materials and their energy band gaps are the deciding factors for the emission wavelengths in LDs. Interestingly, there are some semiconductor compounds in which the bandgap energy can be altered by varying the details of the composition. For example, to achieve shorter wavelengths, one can increase the bandgap energy by increasing the aluminum content (increased x) in AlxGa1-xAs. Table 4 gives a summary of the semiconductor compounds used for developing LDs with different wavelengths. Even though the visible wavelength range is listed here, these are not commonly available in the pulsed mode.

#### 4.2.2. Pulsed Laser Diode Drivers

**Figure 11:**
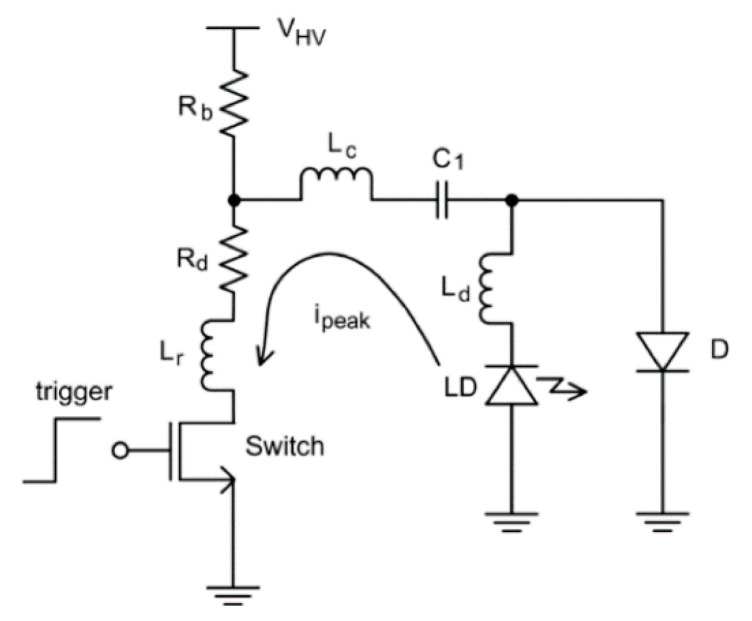
Typical laser driver circuit. Reproduced with permission from H. Zhong, T. Duan, H. Lan, M. Zhou, F. Gao, Sensors, Vol.18, Article ID2264, 2018; licensed under a Creative Commons Attribution (CC BY) license.

Even though continuous wave modulations have also been explored to drive LDs, the most common way is to pulse modulate to generate the short light pulses required for PAI [15,54]. A typical LD driver circuit is shown in Figure 11 and the basic idea of driving is similar to that of an LED driver detailed in Section 3.2.2 (charging and discharging of a capacitor).

**Figure 12:**
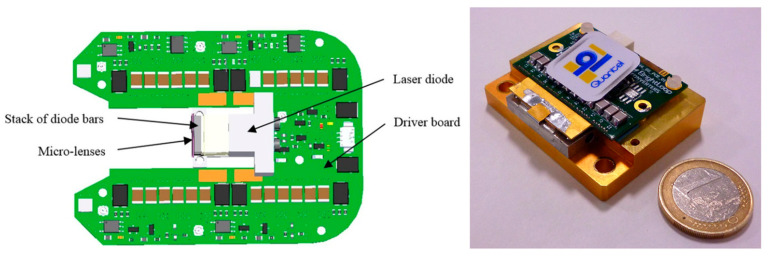
Schematic diagram of the diode laser source showing implementation of the laser diode within the driver board (**left**). An ultra-short pulse laser source integrating a mini-laser diode providing 1.7 mJ in pulses as short as 40 ns from Quantel, France (**right**). Adapted with permission from Proc. SPIE 9887, Biophotonics: Photonic Solutions for Better Health Care V, 98872B (2016). Copyright 2016 Society of Photo-Optical Instrumentation Engineers (SPIE).

The pulse current flows from the LD to the switching device, as illustrated by the arrow in Figure 11. In order to attain the lower pulse width and boost the peak current, the parasitic inductance (L_r_, L_c_ and L_d_) should be as small as possible [15,55]. Usually the time constant for these circuits are fixed and tuning of the pulse width is not an option. It is worth mentioning that LEDs on the other hand has already proven to generate light pulses of different pulse widths (30–100 ns). To the best of our knowledge, the most efficient LD driver in the literature so far was reported in 2016, with a size of 40 × 50 mm^2^, a PRR up to 10 kHz, and a pulse energy of 1.7 mJ per pulse with a pulse width of 40 ns [56]. In this work, Canal et al. reported the possibility of integrating a high power LD in its own driver electronic board to save space (Figure 12) and thus opening up the possibility to develop handheld PA probes.

#### 4.2.3. Optical Output Power

**Figure 13:**
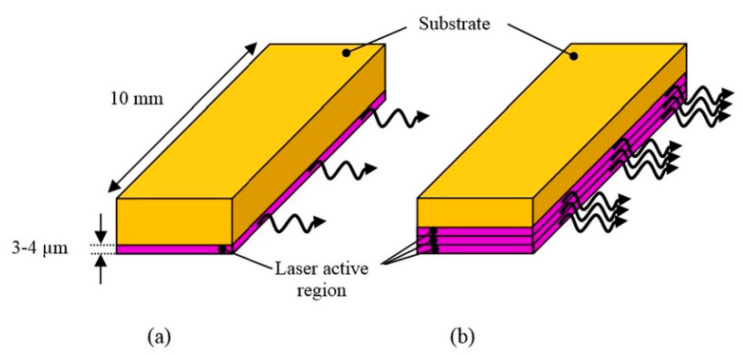
Schematic diagram of (**a**) a standard diode bar and (**b**) a multiple active region diode bar. Adapted with permission from Proc. SPIE 9887, Biophotonics: Photonic Solutions for Better Health Care V, 98872B (2016). Copyright 2016 Society of Photo-Optical Instrumentation Engineers (SPIE).

Conventional pulsed LDs generate laser pulses of relatively low energies over a range of few hundreds of nanojoules to a few microjoules per pulse. However, similar to LEDs, with high PRR LDs (~10 KHz), one can average multiple frames and improve the SNR and imaging depth without much impact on the temporal resolution. Recent developments in semiconductor technology also had positive impacts on the field of LDs. Using diode-stacking technology and ultracompact drivers, a maximum output energy of 1.7 mJ per pulse was reported in 2016 [56]. Combined with a PRR of 10 KHz and a pulse width of 40 ns, these powerful LDs hold strong potential in deep-tissue high-resolution PAI. Figure 13 shows a schematic comparing diode bars with single and multiple active regions.

#### 4.2.4. Pulse Repetition Rate

Typically, pulsed LDs can be driven at repetition rates of 1–10 KHz, which is far higher than the PRR of conventional lasers. To the best of our knowledge, the highest PRR reported for an LD is 10 KHz, which is high enough to be useful for compensating for a low output pulse energy [56]. In this regard, there is flexibility to average multiple frames, maintaining the real-time imaging capability just as in the case of LEDs. Since LDs are coherent light sources as lasers, it is also important to consider the maximum permissible exposure when using higher PRR to ensure light safety, especially in a clinical setting [53].

#### 4.2.5. Pulse Width

Pulse widths of commercially available pulsed LDs are in the range of 30–200 ns [53]. This range is higher than that of a conventional solid-state laser, which is capable of delivering 5–10 ns pulses. However, as discussed before, if stress and thermal confinement can be met, such a low pulse width is not a necessity for deep-tissue PAT. For example, when using 100 ns LDs as an illumination source, the maximum PA signal frequency expected will be around 10 MHz, which is well within the detection bandwidths of US transducers suitable for imaging deep-tissue structures. However, for high-resolution imaging of shallow structures using high-frequency US probes, the pulse widths of the LDs may not be sufficient; also, it is important to mention that the pulse energy of the LD-generated light pulses will drop when the pulse width is reduced. Pulse width-tunable LDs are yet to be reported, which may be because of the technical difficulties in the driver circuit.

#### 4.2.6. Spatial Divergence of LD

The spatial divergence of LDs is larger than that of solid-state lasers but smaller than that of LEDs in general, with angles of up to 40 degrees in the axis perpendicular to the diode arrays (“fast axis”) and 10 degrees in the parallel axis (“slow axis”) [57]. It has been shown that one can efficiently reshape the divergent beam from LDs to be used in an integrated PA and US probe [57]. However, tight optical focusing with these high-power LDs is not feasible due to their multimode nature, which limits their application in optical-resolution PA microscopy.

### 4.3. Technical Developments in LD-Based Photoacoustic Tomography

To the best of our knowledge, the first report on using LDs as a PA illumination source was from Allen and Beard back in 2005 [58]. In this work, they used a pulsed LD (P GAF5S24 from EG&G; wavelength: 905 nm; and pulse duration: 200 ns) driven by a commercially available electronic driver (PCO−7120 from DEI). In this proof-of-concept work, they used a non-realistic phantom (ink-filled cell) and the light was delivered to it using a multimode fiber coupled to the diode. Apart from showing the feasibility of generating PA signals using an LD, they also studied the impact of pulse duration in PAI. In another work immediately after this, Kolkman et al. demonstrated that it was feasible to generate PA signals from superficial blood vessels in a human volunteer [59]. They used a commercially available pulsed laser diode module (iRLS, Laser Components GmbH, Germany) and compared its PAI efficiency with a conventional solid-state laser. The LD used was of 905 nm wavelength with a pulse duration of 112 ns and a PRR of 5 kHz, and offered a maximum optical energy of 23 µJ per pulse. Acoustic detection was performed using a double-ring sensor that can generate 1D depth images (A-scans), which then can be used to generate 2D images by rendering multiple A-lines together. Using this setup, for the first time they demonstrated that LDs could image blood vessels in vivo. However, in this encouraging study, the time for one scan (above 3 min) and the imaging depth (1 mm) was not optimal.

In late 2016, Allen and Beard improved their 905 nm LD-based PAT system and showed that it was feasible to image a tissue-mimicking blood vessel phantom effectively [60]. This work confirmed that LDs can very well be an alternative to solid-state lasers for superficial PA imaging applications. In this system, the maximum PRR was 5 kHz and the pulse width was tunable from 50–500 ns. Pulse width settings of 65 ns (pulse energy: 24 µJ) and 500 ns (pulse energy: 184 µJ) were used to demonstrate how pulse width affects PA image quality because of stress confinement requirements. A 3.5 MHz single-element PZT focused transducer was used for acoustic detection and 5000 PA frames were averaged to generate one image. Even though slightly slow because of limitations in electronics and data transfer, this work was a solid demonstration of LD-based PAT. Key issues to resolve for using LD-based PAT for in vivo applications were pulse energy and time of acquisition and processing.

**Figure 14:**
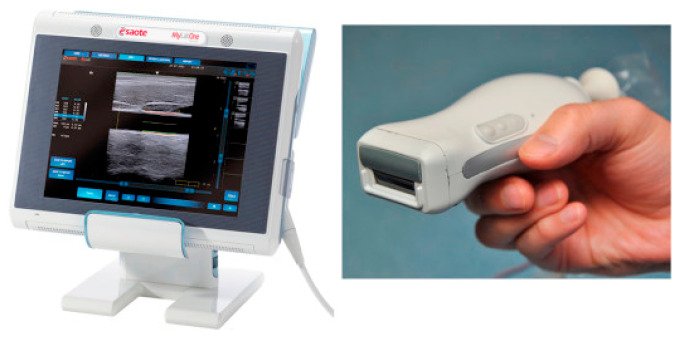
Portable imaging scanner combining photoacoustics and ultrasound. Left is the ultrasound scanner system and right is the picture of the probe integrating the laser module and ultrasound transducer array. Reprinted with permission from K. Daoudi, P.J. van den Berg, O. Rabot, A. Kohl, S. Tisserand, P. Brands, and W. Steenbergen, Optics Express, Vol.22, pp. 26365–26374, 2014; licensed under OSA’s “Copyright Transfer and Open Access Publishing Agreement” (OAPA). Copyright 2014 Optical Society of America.

After these pioneering works on the use of LDs in PAI, there have been significant developments in the field of combining PAI with conventional pulse-echo US imaging. In this regard, by 2014, Daoudi et al. reported an integrated US and PA imaging probe with a high-power LD (Quantel, France) and all optical components integrated inside one housing along with a commercially available 7 MHz linear array US probe (ESAOTE Europe) [57]. The wavelength of the LD was 805 nm, which emitted 130 ns pulses with an optical energy of 0.56 mJ per pulse. The PRR was 10 kHz, opening up the possibility to average multiple frames to improve the SNR without sacrificing the frame rate. Figure 14 shows a photograph of this dual-modality PA and US system. As one can see, the probe is quite portable and well suited for a real-time handheld operation in a clinical setting.

**Figure 15:**
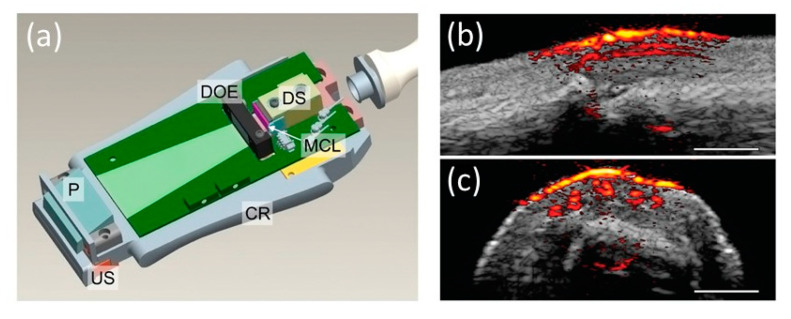
(**a**) A schematic of the handheld PA and US probe. US: ultrasound array transducer; P: deflecting prism; DOE: diffractive optical elements; DS: diode stack; MCL: micro-cylindrical lenses; CR: aluminum cooling rim. Photoacoustic/ultrasound images of a human proximal interphalangeal joint in the (**b**) sagittal and (**c**) transverse planes. Adapted with permission from K. Daoudi, P.J. van den Berg, O. Rabot, A. Kohl, S. Tisserand, P. Brands, and W. Steenbergen, Optics Express, Vol.22, pp. 26365–26374, 2014; licensed under OSA’s “Copyright Transfer and Open Access Publishing Agreement” (OAPA). Copyright 2014 Optical Society of America.

A key feature of this probe design is that all optical components, including the deflecting prism, diffractive optical elements, diode stack, micro-cylindrical lenses, and aluminum cooling rim, were packaged inside a single casing along with a 128-element US probe (Figure 15a). Along with reporting the design of the probe, they performed a detailed characterization of the system and demonstrated the feasibility of in vivo real-time imaging on a human finger. An imaging depth of 4 mm was achieved in the human finger measurement at a frame rate of 20 Hz (Figure 15b,c). Higher imaging depths (10–15 mm) were achieved in phantom studies, but at a higher PRF, which cannot be used in human experiments because of laser safety regulations. Radiant exposure of 1.3 mJ/cm^2^ on the skin with an illumination spot size of 18.2 × 2.3 mm^2^ was achieved in this prototype, resulting in the possibility of imaging micro-vasculature with unprecedented contrast and resolution. The same system was later used for multiple preclinical applications and early clinical pilot studies [61,62,63,64,65,66]. Adding multiple diode stacks with different wavelengths can make this system a power tool with excellent structural, functional, and molecular imaging capability.

**Figure 16:**
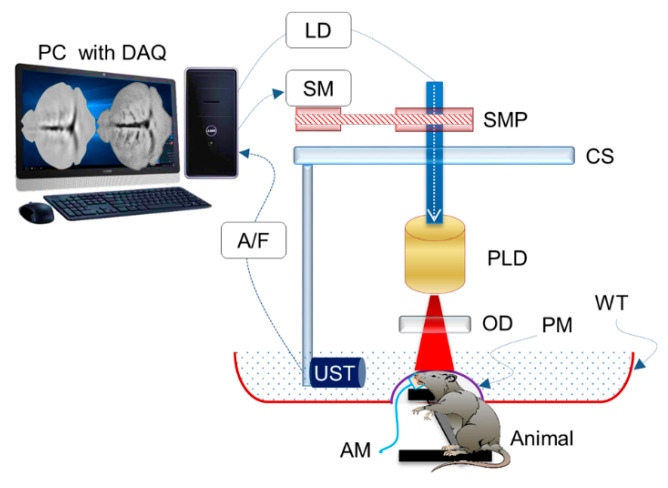
Schematic of the PLD-PAT system for in vivo small animal brain imaging: PLD, pulsed laser diode; OD, optical diffuser; CS, circular scanning plate; SMP, stepper motor pulley unit; UST, ultrasound transducer; A/F, amplifier/filter unit; LD, laser driver unit; SM, stepper motor; PC, personal computer; WT, water tank; DAQ, data acquisition card; AM, anesthesia machine; PM, transparent polythene membrane. Reproduced with permission from P. K. Upputuri and M. Pramanik, Journal of Biomedical Optics, Vol.22, Article ID090501, 2017; licensed under a Creative Commons Attribution (CC BY) license.

**Figure 17:**
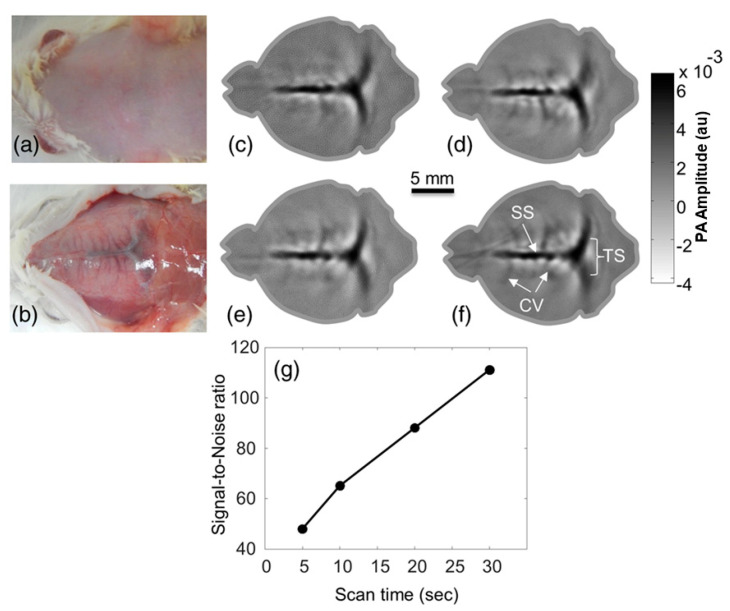
Images of brain vasculature in a 95 g female rat acquired non-invasively with a laser diode-based photoacoustic tomography system at different scan times: photograph of rat brain before (**a**) and after (**b**) removing the scalp. In vivo brain images at a (**c**) 5-s, (**d**) 10-s, (**e**) 20-s, and (**f**) 30-s scan time. (**g**) SNR of the in vivo images as a function of scan time. SS, sagittal sinus; TS, transverse sinus; CV, cerebral veins. Adapted with permission from P. K. Upputuri and M. Pramanik, Journal of Biomedical Optics, Vol.22, Article ID090501, 2017; licensed under a Creative Commons Attribution (CC BY) license.

In late 2015, Upputuri et al. demonstrated the possibility of PAT using LD excitation and scanning of a single-element US transducer [67]. Using a powerful LD from Quantel (wavelength: 803 nm; pulse energy: 1.4 mJ; and PRR: 7 KHz) and a conventional single-element US transducer rotating around the object, they achieved to obtain an image every 3 s and they also demonstrated an imaging depth of 2 cm in phantom studies, which is commendable. They also compared the results with an OPO-based laser system in similar settings. This system was upgraded by them with multiple single-element transducers and used in multiple preclinical applications recently [68,69,70]. Figure 16 shows a schematic of the PLD-PAT system designed for in vivo small animal brain imaging and Figure 17 shows images of brain vasculature in a 95 g female rat acquired non-invasively with PAT at different scan times.

Out of these PLD-PAT works, a recent study from Rajendran et al. [69] is commendable. In this work, the authors used their LD-PAT setup for detecting changes in the sagittal sinus due to intra-cranial hypotension in a rat model. A key advantage of this LD-PAT setup is the cost reduction because of (1) not using US probes with multiple elements; (2) not using multichannel DAQ systems; and (3) replacement of bulky and expensive lasers with LDs. In another recent work from the same group, Upputuri et al. [71] demonstrated a pulsed LD-based PA temperature sensing system for monitoring tissue temperature in real time. The system takes advantages of a laser diode with a high repetition rate (7000 Hz), a near-infrared wavelength (803 nm), and a relatively high energy (1.42 mJ/pulse). Results gave a direct confirmation that this LD-based PAI system is capable of providing local temperature information at a high temporal resolution of 1 ms and high sensitivity of 0.31 °C.

Even though no LD-based PAT system is available commercially, several research studies (Table 5) have validated the potential of LD-based PAT in multiple preclinical and clinical applications, in which encouraging imaging depths were achieved despite low pulse energies when compared with lasers. Pulsed LDs are powerful, portable, and cost-effective, and it is believed that LD-based PAT could be a complementary modality to conventional clinical US imaging [72].

## 5. Discussion

In this review, we focused on the basics of PAI and the use of affordable light sources (LEDs and LDs) as illumination sources in PAT. After a short introduction to the physical phenomenon behind biomedical PAI, we elaborated on the key specifications and technological developments in the area of high-power LEDs and LDs and detailed their use as illumination sources in PAT. It is encouraging that even with low optical energies, LED and LD-based PAT systems have already demonstrated their potential in a wide range of functional (oxygen saturation imaging, blood flow imaging, etc.) and molecular imaging applications (tracking contrast agents, pharmacokinetic studies, etc.), thanks to the high PRR (maximum reported PRR for LED: 16 KHz; LD: 10 KHz) and possibility to average over a large number of frames to improve the SNR [13,14,15]. With these affordable light source-based PAT systems, several in vivo preclinical and clinical pilot studies have reported imaging depths of above 8–10 mm, at frame rates unachievable for conventional laser-based systems [13,14,15]. Apart from the technical aspects in developing an LED and LD-based PAT system, we also shortly introduced the wide variety of preclinical applications and clinical pilot studies reported using these. Divergence of the LEDs and their large beam size when using an array of LDs make these semiconductors not the optimum choice for PA microscopy applications where a tight light focus is an important requirement [23]. However, there are some reports on the use of LEDs and LDs for microscopic and shallow-depth imaging applications, too [74,75,76,77,78,79]. We did not include these details in this review as our main focus was on tomographic setups and applications.

PAI is already matured in the research setting and has demonstrated its unprecedented potential in a wide range of biomedical imaging applications. However, the clinical translation of this promising technology is not happening at the expected pace. One of the important reasons for this is the requirement of bulky, slow, and expensive pulsed lasers. In the last decade, there have been significant developments in the semiconductor device technology and use of portable and affordable devices like LEDs and LDs became popular as pulsed light sources in PAT. Q-switched solid-state lasers with water-cooling are indeed not an optimal choice for use in a clinical setting because of their sizes, power consumption, costs, and also skin/eye safety aspects (users and patients must wear eye safety glasses and systems must be installed in a laser-safe room). The PRRs of these lasers are also slow and real-time imaging can be challenging, especially when frame averaging is a requirement for improving SNR. Considering the portability, affordability, and dynamic imaging capability aspects, both LEDs and LDs hold strong potential in replacing solid-state lasers, especially for superficial and sub-surface imaging applications, such as in rheumatology, dermatology, and cardiovascular medicine. Handheld US imaging is one of the most popular medical imaging modalities and it is seamless to implement PAT in conventional clinical pulse-echo US equipment. In such handheld systems, integration of LEDs and LDs in a single housing along with a US probe will be a straightforward development and an important aspect to consider in these integrated probes is the mechanism for absorbing the heat generated because of high-speed pulsing. In terms of eye and skin safety, LDs with its coherent nature is similar to that of a conventional laser and the requirements of eye-safety goggles and laser-safe rooms remain important. Even though the fundamental light generation processes for LEDs and LDs are similar, LEDs do not generate stimulated emissions and they possess a broad optical bandwidth and their spatial coherence is low. Considering these factors, LEDs are not practically considered as laser sources and thus could be potentially used as an eye/skin-safe light sources for PAI given the low optical fluence, and the system could be installed in non-laser safe rooms, too, making it an ideal choice for using in a resource-limited clinical setting [13].

Even though LEDs and LDs have shown their potential in biomedical PAT, the pulse energy of these diodes is still a bottleneck when compared to a solid-state laser, and this limits their use only to superficial imaging applications. The maximum pulse energy reported by an LED source (when used in an array form) is 200 µJ at an 850 nm wavelength. When two such arrays are used on both sides of an US probe, the total light output will be 400 µJ per pulse. In such a setting, the maximum imaging depth achieved in vivo was reported to be around 10 mm, with a combined US and PA frame rate above 10 Hz. On the other hand, LDs are more powerful and a pulse energy above 2 mJ is achievable. However, in an animal/human in vivo situation, so far no LD-based PAI studies has reported an imaging depth above 5–6 mm. An exception is the study from Jaeger et al., in which a carotid artery at a depth of 15 mm was visualized using an integrated probe with an 808 nm LD and a 7 MHz linear array probe. This was achieved by applying an algorithm called DCA (deformation compensated averaging) to reduce the clutter and thereby improving the SNR. It is not straightforward to use a clinical US system for PA detection as highly sensitive and broadband detection with high amplification is very important to achieve high imaging depths. In a nutshell, the maximum imaging depth currently achievable using LED and LED-based PAI systems is less than 2 cm, making it difficult to target deep-tissue imaging applications. Imaging depth in PAI is dependent on several factors, including illumination (type of illumination source, optical output power, wavelength, illumination area, etc.), acoustic detection strategies (sensitivity, directionality, spatial distributions of the US detectors, and noise performance of DAQ, etc.), and image processing and reconstruction algorithms. The conventional solid-state laser-based PAT has already achieved an excellent imaging depth above 6 cm in phantom studies and 3–4 cm in in vivo clinical pilot studies [80]. It is worth mentioning that a higher imaging depth (4 cm) was achieved using curved US probes in a computed tomographic setup [81,82]. Using clinical linear array US probes, to the best of our knowledge, an imaging depth above 2 cm has not been demonstrated in vivo even when using bulky and powerful solid-state lasers as illumination sources. We believe that further advancements in semiconductor device technology and lighting industry in general will improve the pulse energy offered by LDs and LEDs in the near future, thus making these devices well-suited for PAT applications. Theoretically, it is feasible to improve the SNR and thereby imaging depth by increasing the PRR and thus offering more room to increase frame averaging. However, heat generation (especially in fully integrated US/PA probes) and also laser safety will be serious concerns when the PRR is extremely high. It is also well known that when the N frames are averaged, the SNR will be improved only by √N. Several US-received side strategies and AI-based methods have also been reported for improving the image quality [65,83,84,85,86,87,88,89,90,91]. We foresee that developments in the area of semiconductor devices and low noise electronics, combined with advanced image reconstruction and enhancement algorithms, will accelerate the clinical translation of affordable light sources based PAI. Since SNR is a key problem to solve, use of novel coded excitation schemes [24,92] and implementation of clutter and reflection artifact reduction algorithms [65,93] also will have a significant impact in clinical translation of LED and LD-based PAT. When compared to OPO-based lasers, one of the key disadvantages of LEDs and LDs is the difficulty in obtaining multiple illumination wavelengths for multispectral PAI [13]. When multiple diode elements with different wavelengths are embedded in an arrayed form, the pulse energy will be further reduced, which then has a serious impact on the imaging depth. Dual-wavelength LED-based PAT for applications like oxygen saturation imaging and differentiation of veins and lymphatic vessels are already reported [25,40]. However, in a complex situation with more than two optical absorbers in the tissue, OPO-based laser systems are still preferred because of the wide range of wavelengths available and fast tuning capability.

The pulse duration of the excitation light source is one of the key factors that has an impact on PA signal generation and image quality. It is well known that a short pulse width is important for satisfying the stress confinement criteria and generating broadband PA signals suitable for high-resolution images. Typical pulsed lasers offer a pulse width in the range of 3–10 ns, which then can generate broadband PA signals from the optically absorbing objects. For example, a 3.5 ns light pulse can generate PA signals with frequencies up to 300 MHz, depending on the size of the targets. Such high frequencies from deep tissue will be heavily attenuated and will not reach the US probe. If one has to detect such signals from superficial tissue, it is important to use high frequency US probes with a super-high reception bandwidth. Even though this is feasible technically, applications will be limited to PA microscopy. When using a conventional mid-frequency US probe suitable for deep tissue imaging, the US detection efficiency with frequencies above 12–14 MHz is extremely low [13]. Considering these factors, we believe that such a low pulse width is not an absolute necessity, provided that the stress confinement is met. As discussed before, a very low pulse widths will be only interesting for PA microscopy imaging applications with imaging depth less than 1–2 mm.

In this review, we mainly focused on LEDs and LDs as these are the most common and well-explored affordable PAT light source, especially in the NIR wavelength range, which is the most suitable range of light wavelengths for deep tissue imaging applications [14,15,53]. However, in recent years there have been several other reports too on affordable illumination strategies in PAT. In 2016, Wong et al. reported on the use of a single Xenon flash lamp as a light source in PAT [94]. In this promising work, the PRR of the Xenon lamp could be controlled between 10–100 Hz and 3 mJ of energy was carried in pulses with a 1 μs pulse width. Using a 0.5 MHz ring-shape detector, the authors demonstrated the feasibility of the imaging tissue, mimicking phantoms (imaging depth: 3.5 cm) and a whole mouse body in vivo. Recently, there have been promising reports on the use of diode-pumped solid-state lasers (DPSSLs) as a light source in PAT. PDSSLs are portable and energy efficient, and thus a promising light source for PAI in a resource-limited setting. Wang et al. used a compact high-power DPSSL (Montfort Laser GmbH Inc., Germany) for deep tissue single wavelength in vivo PAT imaging [95]. This laser source has a miniature size of 13.2 × 14.0 × 6.5 cm^3^, a weight of 1.6 kg, and an average power output of 4 W, with a high pulse energy of up to 80 mJ at the wavelength 1064 nm with a PRR up to 50 Hz. Using this DPSSL-based system, the authors successfully imaged murine whole-body vascular structures and cardiac functions in vivo, and mapped the arm, palm, and breast vasculatures of living human subjects. One of the key advantages of these DPSSLs are the possibility of fiber coupling and consequent focusing, which is very much important for PA microscopy imaging applications (this is not possible using LED and LEDs). Recently, Jeng et al. reported on the use of a compact 700–900 nm tunable DPSSL with a pulse energy of around 1 mJ over the range of wavelengths and a PRR of 1 KHz [96]. In this work, the authors demonstrated automatic laser-fluence compensation in spectroscopic PAT and inter-wavelength motion correction using US speckle tracking, which has never been shown before in real-time systems. The 50-Hz video rate PAUS system was demonstrated in vivo using a murine model of drug delivery monitoring. As an energy-efficient and portable illumination source, pulsed fiber lasers were also explored as a PAT illumination source. Using a fiber laser emitting at 1060 nm, with a maximum pulse energy of 0.8 mJ and a Fabry Perot ultrasound scanner, Allen et al. demonstrated the potential of 3D PAT with a realistic blood vessel phantom and the palm vasculature of a human volunteer [97]. Frequency domain PAI systems using continuous-wave modulated laser diodes and LEDs have also been developed recently for several biomedical applications [98,99].

Since short-pulsed light can have a serious impact on the eyes/skin, it is very important to keep the optical pulse energy within the maximum permissible exposure (MPE) limit by following, e.g., the American National Standards Institute (ANSI). Even though the LEDs used in PAI generate pulsed light, they are not considered as laser sources and at present there are no safety standards defined in this regard, to the best of our knowledge. Light from LEDs is optically broad and non-coherent, making it considerably safer to use in a clinical setting as compared to lasers in general (divergence of LEDs is also high, and no safety issues are expected unless the light source is kept very close to the eyes). As a high PRR pulsed laser source, it is very important to consider the MPE limits of the LDs. One may get an initial impression that portable devices like LDs with a low optical power will be completely safe for the eyes and skin. Unfortunately, this is not the case because the light density on the skin will be high when the repetition rate is very high (a large number of light pulses over a period of time). ANSI has clear definitions for this and the safety limits for the skin depend on the light wavelength, pulse width, duration of the exposure, and illumination area [51]. For example, let us consider the case of 800-nm wavelength (λ), the MPE limit for the skin over an exposure time (t) of 0.5 s (assumption) is given by 1.1 × 10^(2(λ−700)/1000)^ × t^0.25^ J/cm^2^ = ~1.47 J/cm^2^. For an LD with a PRR of 2 KHz, the MPE per pulse becomes ~0.735 mJ/cm^2^ (1.47/2000). If the LD can deliver optical energy of 1 mJ per pulse, the light beam must be expanded in such a way that the total area of illumination is about 1.36 cm^2^ (1/0.735). From this, it is clear that for increasing the PRR for the same exposure time of 0.5 s, the MPE per pulse must be lower than 0.735 mJ/cm^2^. In a nutshell, the total pulse energy density will be lower when one increases the PRR, subsequently reducing the possibility of averaging and thus SNR reduction. In clinical PAI, it is of paramount importance to carefully select the pulse energy, PRR, illumination area, and time of exposure for keeping up with the MPE safety limits [52].

## 6. Conclusions

Compact, fast, affordable, and energy-efficient light sources are important requirements for accelerating the clinical translation of PAI. LEDs and LDs fit very well into this category and have been explored extensively in recent years as an illumination source in PAI. This review, which is focused on affordable light source-based PAI, is timely considering the fact that this promising technology is facing an exciting transition from bench to bedside.

In this paper, we first covered the basic theory of PAI, clearly conveying the key advantages of the technique, including optical contrast and acoustic resolution/imaging depth. In the first section, we also shortly introduced the different light sources used in PAI with its specifications, including pulse energy, PRR, pulse width, and cost. After this, the principles of light generation and basic performance characteristics of both LEDs and LDs were discussed with a focus on the advantages and disadvantages when they are used as a light source in PAI. The historical developments (from single-point measurements to in vivo imaging and to commercialization) of both LED- and LD-based PAI systems were also discussed, listing out the key preclinical and clinical application demonstrations.

Even though LEDs and LDs possess some disadvantages, such as low optical energy, lack of spectral tuning capability, and long pulse widths, they are portable, affordable, and energy-efficient light sources. Apart from point-of-care biomedical imaging, LEDs and LDs would be an ideal choice for wearable PA equipment and may find a plethora of applications in the field of therapeutic drug monitoring. Integration of LED- or LD-based PAI to a clinical US scanner will have an easier clinical acceptance when compared to laser-based PAI. We expect that dual-mode US and PA equipment utilizing affordable light sources will have a significant impact on bedside diagnostic imaging, accelerating the translation of this technology from research labs to clinics.

## Figures and Tables

**Table 1:** Comparison of different light sources used in photoacoustic imaging. * Cost includes the driving electronics and may vary based on different features, number of wavelengths, etc. Integration to a US probe may also involve extra development cost. LD, Laser diode; LED: Light-emitting diode; DPSS: Diode-pumped solid-state; PRR, pulse repetition rate. Adapted with permission from Y. Zhu, T. Feng, Q. Cheng, X. Wang, S. Du, N. Sato, J. Yuan and M. Kuniyil Ajith Singh, Sensors, Vol.20, Article ID2484, 2020; licensed under a Creative Commons Attribution (CC BY) license.

	Energy (mJ)	PRR (Hz)	Pulse Width (ns)	Cost *	Advantages	Disadvantages
Solid-state lasers	5–120	10–200	<10	$70–200 K	Powerful, ~5 cm penetration depth, tunable wavelength	Bulky size, eye protection and laser safe rooms needed
LD	0.5–2.5	~1 K–6 K	30–200	~$10–25 K	Integration in a handheld probe feasible, high PRR	Limited penetration depth, eye protection and laser safe rooms needed, wavelength tuning not possible
LED	0.2	~200–16 K	30–100	$10–15 K	Integration in a handheld probe feasible, high PRR, wide wavelength range, no need of laser-safe rooms and eye-safety goggles	Limited penetration depth, wavelength tuning not possible
Q-switched DPSS laser	1	100 K	2–10	-	High PRR, low pulse width, Reasonably high optical energy per pulse	Less number of wavelengths (266 nm, 355 nm, 532 nm, 1064 nm) available and spectral tuning may be cumbersome
High-energy DPSS laser	200	200	10–30	-	High optical output per pulse, reasonably high PRR	Less number of wavelengths (266 nm, 355 nm, 532 nm, 1064 nm) available and spectral tuning may be cumbersome

**Table 2:** Summary of the materials used to design LEDs with different wavelengths.

Wavelength (nm)	440–550	570–650	624–920
Material	InGaN	AlGaInP	AlGaAs

**Table 3:** Summary of the preclinical and clinical applications of LED-based PAI. Adapted with permission from Y. Zhu, T. Feng, Q. Cheng, X. Wang, S. Du, N. Sato, J. Yuan and M. Kuniyil Ajith Singh, Sensors, Vol.20, Article ID2484, 2020; licensed under a Creative Commons Attribution (CC BY) license. ICG, indocyanine green.

Target	Application		Depth (mm)	Contrast Agent	Wavelength (nm)
Medical needles, Vasculature	Guidance of minimally invasive procedures with peripheral tissue targets [18]	Phantom and ex vivo studies	38	N/A	850
Vasculature	Imaging of human placental vasculature [48]		7	N/A	850
Tumor	Imaging of intraocular tumors [26]		10	N/A	850
Vasculature	Non-invasive monitoring of angiogenesis [51]	Animal in vivo	10	N/A	850
Ulcer	Noninvasive imaging of pressure ulcers [47]		10	N/A	690
Oxygen saturation	Oxygen saturation imaging in rheumatoid arthritis [39]		5	N/A	750/850
Molecular	Detection and monitoring of reactive oxygen and nitrogen species [49]		10	CyBA	850
Tumor/Contrast agents	Imaging of tumor using contrast enhancement [44]		10	NC	850
Cells/Contrast agents	Imaging of molecular-labelled cells [38]		10	DiR	850
Vasculature	Imaging of peripheral microvasculature and function [26]	Healthy human	10	N/A	690/850
Vasculature	Simultaneous imaging of veins and lymphatic vessels [40]		10	ICG	940/820
Finger joints	Full view tomography of finger joints [28]		5	N/A	850
Finger joints	Imaging of inflammatory arthritis [42]	Patient	5	N/A	850
Skin	Imaging of port wine stain [43]		10	N/A	850

**Table 4:** Summary of the materials used to design LDs with different wavelengths.

Wavelength (nm)	630–670	720–850	900–1100
Material	AlGaInP/GaAs	AlGaAs/GaAs	InGaAs/GaAs

**Table 5:** Summary of the preclinical and clinical applications of LD-based PAI. ICG, indocyanine green.

Target	Application		Depth (mm)	Contrast Agent	Wavelength (nm)
Vasculature	Detection of intraplaque hemorrhage in carotid artery [62]	Phantom and ex vivo studies	20	N/A	808
Vasculature	Dynamic imaging studies (for example in cardiovascular medicine) [73]		20	N/A	803
Red blood cells	Non-invasive blood flow imaging [66]		7	N/A	805
Vasculature	Detection of liver fibrosis [64]	Animal in vivo	5	N/A	808
Cortical vasculature	Brain imaging [68]		5	ICG	803
Cerebro-spinal fluid volume level	Detection of venous sinus distension by measuring intra-cranial hypertension [69]		5	N/A	803
Vasculature and perfusion	Vascular/dermal pathologies [63]	Healthy human	5	N/A	805
Vasculature	Detection of intraplaque hemorrhage in carotid artery [65]		15	N/A	808
Finger joints	Imaging of rheumatoid arthritis [57]		5	N/A	808
Finger joints	Imaging of rheumatoid arthritis [61]	Patient	5	N/A	808
